# Assessment of the Safety and Efficacy of an Oral Probiotic-Based Vaccine Against Aspergillus Infection in Captive-Bred Humboldt Penguins (*Spheniscus humboldti*)

**DOI:** 10.3389/fimmu.2022.897223

**Published:** 2022-05-13

**Authors:** Milan Thorel, Lourdes Mateos-Hernandez, Baptiste Mulot, Mouna Naila Azzouni, Adnan Hodžić, Hugues Gaillot, Yannick Ruel, Guillaume Desoubeaux, Jean-Baptiste Delaye, Dasiel Obregon, Alejandra Wu-Chuang, José de la Fuente, Luis G. Bermúdez-Humarán, Veronica Risco-Castillo, Antoine Leclerc, Alejandro Cabezas-Cruz

**Affiliations:** ^1^ ZooParc de Beauval and Beauval Nature, Saint-Aignan-sur-Cher, France; ^2^ Anses, INRAE, Ecole Nationale Vétérinaire d’Alfort, UMR BIPAR, Laboratoire de Santé Animale, Maisons-Alfort, France; ^3^ Department of Pathobiology, Institute of Parasitology, University of Veterinary Medicine Vienna, Vienna, Austria; ^4^ ADVETIA Veterinary Hospital Center, Vélizy-Villacoublay, France; ^5^ CHU de Tours, Service de Parasitologie, Mycologie, Médecine Tropicale, Tours, France; ^6^ Université de Tours, Inserm U1100 – Centre d’Etude des Pathologies Respiratoires, Faculté de Médecine, Tours, France; ^7^ CHU de Tours, Pôle de Biologie médicale, Laboratoire de Médecine Nucléaire In Vitro – Centre Régional de Dépistage Néonatal, Tours, France; ^8^ School of Environmental Sciences, University of Guelph, Guelph, ON, Canada; ^9^ SaBio, Instituto de Investigación en Recursos Cinegéticos IREC-CSIC-UCLM-JCCM, Ciudad Real, Spain; ^10^ Department of Veterinary Pathobiology, Center for Veterinary Health Sciences, Oklahoma State University, Stillwater, OK, United States; ^11^ Université Paris-Saclay, INRAE, AgroParisTech, Micalis Institute, Jouy-en-Josas, France; ^12^ EA 7380 Dynamyc, UPEC, USC, ANSES, Ecole nationale vétérinaire d’Alfort, Université Paris-Est, Maisons-Alfort, France

**Keywords:** α-Gal Vaccine, aspergillosis, *Aspergillus fumigatus*, probiotics, *E. coli* Nissle 1917, penguins, *Spheniscus humboldti*, zoo

## Abstract

Aspergillosis is a fungal infection caused mainly by *Aspergillus fumigatus* that often results in respiratory disease in birds. Aspergillosis is a major cause of morbidity and mortality in captive-bred penguin species. Currently, there is no registered vaccine to prevent aspergillosis. Recent research demonstrated that oral administration of gram-negative bacteria expressing high levels of galactose-α-1,3-galactose (α-Gal) modulates anti-α-Gal immunity and protects turkeys from clinical aspergillosis caused by experimental *A. fumigatus* infection. The role of anti-α-Gal immunity in penguins has not been studied. Here, we tested the distribution of α-1,3-galactosyltransferase (α1,3GT) genes in the fecal microbiome of Humboldt penguins (*Spheniscus humboldti*). The occurrence of natural anti-α-Gal antibodies (Abs) in sera and eggs of healthy Humboldt penguins was also assessed. A trial was then conducted to test whether oral administration of *Escherichia coli* Nissle, expressing high α-Gal levels, modulates anti-α-Gal immunity in a colony of Humboldt penguins. Animals in the vaccination and placebo groups were evaluated before the trial and followed for one year for aspergillosis detection using a diagnostic panel including computed tomography scans, capillary zone electrophoresis, 3-hydroxybutyrate levels, and anti-*A. fumigatus* Abs. Anti-α-Gal Abs were detected in sera (IgM and IgY) and eggs (IgY) of healthy penguins. Microbiota analysis and functional predictions revealed the presence of α1,3GT genes in the microbiota of Humboldt penguins and other penguin species. A strong decrease in anti-α-Gal IgM levels was observed in all animals in the placebo group three months after vaccination protocol. This decrease was not observed in *E. coli* Nissle-treated penguins. After the vaccination protocol, we found a positive correlation between anti-*E. coli* IgY and anti-α-Gal IgY in the *E. coli* Nissle group, suggesting a correlation between the presence of the bacteria and these Abs. During the study period, three penguins exhibited respiratory signs consistent with aspergillosis. Two were from the placebo group whose symptoms resolved with specific treatments, while a single vaccinated individual developed fatal respiratory aspergillosis eight months after the trial. We conclude that *E. coli* Nissle represents a safe potential probiotic with a protective effect against aspergillosis in Humboldt penguins that deserves to be further explored for therapeutic uses in these animals.

## Introduction

Aspergillosis is an infectious disease caused by the ubiquitous opportunistic saprophytic fungi from the genus *Aspergillus*, primarily *Aspergillus fumigatus* (1). Inhalation of fungal spores is the main route of infection in birds (2). When the bird’s immunity is unable to address the infection with too many spores being inhaled, clinical presentation varies from acute cases involving the lungs to chronic, disseminated infections of the air sacs (1). Mycelial granulomas called aspergillomas may develop within the lungs or other organs, as well as fungal necrotizing plaques resulting in thickening of avian air sac mucosa (3), both of which may obstruct the respiratory airways. Predisposing factors in birds include immunosuppression causes, genetic factors, nutritional (hypovitaminosis A), or infectious and toxic disorders (4, 5). Penguins are among the most affected captive-bred bird species (6–14).

Yearly fatal *A. fumigatus* cases affect approximately 10% of the penguin colony at ZooParc de Beauval in France (personal communication from author MT) and can represent 27% of all cause-mortality in some penguin colonies (12). Fungal loads in their environment settings are often quite high (15), making immunosuppressed or stressed individuals, particularly at risk of developing clinical aspergillosis (2), especially during the summer season (July-September) in the northern hemisphere (13, 15). Diagnosis in birds is challenging, and often requires a multi-tool diagnostic panel in addition to the anamnesis and clinical evaluation (16, 17). Treatment of aspergillosis in clinically infected penguins primarily relies on azole drug-based therapy (18–20). Alternatives include terbinafine (21), amphotericin B (22), or other antifungals. However, clinical signs are non-specific, the diagnosis in the laboratory is challenging, and all together the initiation of adequate antifungal therapy is consequently often delayed. Besides, voriconazole toxicity has been described in penguin species (23) and resistance of *Aspergillus* sp. strains to azole molecules is reported from low (24, 25) to high (8) depending on the zoological institutions. For all these reasons, medical prophylaxis against aspergillosis is paramount in penguins.

The glycan galactose-α-1,3-galactose (α-Gal) is expressed on the surface of several pathogens including *Plasmodium* spp (26)., *Trypanosoma cruzi* (27, 28), *Leishmania* spp. (29), and *A. fumigatus* (30), as well as on glycoconjugates in cells of non-primate mammals, prosimians, and New World monkeys. However, birds, fish, and humans evolved with the inability to synthesize the α-Gal due to the functional inactivation of the α1,3-galactosyltransferase (α1,3GT) gene. In turn, they can produce high levels of anti-α-Gal antibodies (Abs) in response to antigenic stimulation from gut microbiota bacteria, which may be protective against multiple α-Gal-containing pathogens (31). The protective role of anti-α-Gal immunity has been validated in several infectious models, as immunization with the glycan protects against infection with *Plasmodium* spp (26)., *T. cruzi* (32), and *Leishmania* spp (27). Notably, oral administration of the bacterium *Escherichia coli* O86:B7 with high α-Gal content elicited an anti-α-Gal Abs response associated with protection against malaria transmission in α1,3GT-deficient mice (26). In another study, probiotic formulations including *Aeromonas veronii* and *Pseudomonas entomophila* with high content of α-Gal were biosafe and increased the levels of anti-α-Gal IgM and protected against *Mycobacterium marinum* in a zebrafish model (33). Protective mechanisms that regulated immunity and metabolism activated in response to probiotics included modification of the gut microbiota composition, B-cell maturation, anti-α-Gal antibodies-mediated control of mycobacteria, while they induced innate immune responses, beneficial effects on nutrient metabolism, and reduced oxidative stress (33). In addition, recent research showed that oral administration of *Escherichia coli* O86:B7 prevented turkeys from developing aspergillosis after experimental infection with *A. fumigatus* (30). However, the protective effect of *E. coli* O86:B7 was not associated with an increase in circulating anti-α-Gal IgY levels, but with a striking reduction of anti-α-Gal IgA in the lungs of infected turkeys (30). Animals treated with *E. coli* O86:B7 experienced lower lung inflammation, which was associated with no activation of pro-inflammatory cytokine genes (i.e., *IFNγ*, *IL6*, *IL2*) in *A. fumigatus*-infected turkeys. In the light of this evidence, probiotic-based vaccines were proposed as an alternative to activate protective mechanisms associated with the α-Gal immunity for the control of infectious diseases in fish, birds, and humans (31, 34–36).

Developing an anti-α-Gal oral vaccine opens the perspective to protect penguins against various infectious diseases, including malaria, mycobacteriosis, and aspergillosis. However, little is known about α-Gal immunity in Sphenisciformes. In this study, we first studied the occurrence of anti-α-Gal Abs in healthy Humboldt penguins as well as in penguins with clinical suspicion of aspergillosis and in penguin eggs. Then, the presence and abundance of α1,3GT genes were inferred from the 16S rRNA data using the bioinformatics pipeline PICRUSt2 for metagenome prediction. We also tested the safety of using oral administration of *E. coli* Nissle with high content of α-Gal to modulate the anti-α-Gal immunity in penguins. The results advance our current knowledge about α-Gal immunity and show that *E. coli* Nissle is a safe probiotic to use in penguins with protective potential against aspergillosis to be further studied in this species.

## Materials and Methods

### Penguins Gut Microbiome Analyses

#### Feces Collection in Humboldt Penguins, DNA Extraction and 16S rRNA Sequencing

During manual restraint for annual avian influenza vaccination, cloacal swabbing was performed on four Humboldt penguins from the study colony (randomly selected). Genomic DNA was extracted from cloacal samples using Nucleospin tissue DNA extraction Kit (Macherey-Nagel, Hoerdt, France). The samples were deposited inside an extraction buffer kit to remove fecal material, and the manufacturer’s instructions were followed to purify the genomic DNA. Fecal genomic DNA was eluted in 100 µl of sterile water and its quality (OD260/280 between 1.8 –2.0) was measured with NanoDrop™ One (Thermo Scientific, Waltham, MA, USA). More than 200ng of DNA at ≥ 20 ng/µL concentration were sent for amplicon sequencing of the bacterial 16S rRNA gene, which was commissioned to Novogene Bioinformatics Technology Co. (London, UK). Libraries were prepared with NEBNext^®^ Ultra™ IIDNA Library Prep Kit (New England Biolabs, MA, USA). A pair lane of Illumina MiSeq system was used to generate 251-base paired-end reads from the V4 variable region of the 16S rRNA gene using barcoded universal primers (515F/806R). The emerged raw 16S rRNA sequences obtained from cloacal penguin samples were deposited at the SRA repository (Bioproject No. PRJNA808259).

In addition to *S. humboldti*, the presence of α1,3GT genes was also predicted in the gut microbiome of other penguin species including *Eudyptes chrysolophus*, *Eudyptula minor*, *Pygoscelis papua*, and *Aptenodytes patagonicus* [Bioproject PRJEB3083 (37)].

#### 16S rRNA Sequences Processing

After downloading from SRA repository (38), the raw 16S rRNA sequences were uploaded and analyzed using the QIIME2 software (v. 2021.4) pipeline (39). The fastq files were first demultiplexed and filtered for quality control using DADA2 pipeline (40), implemented in QIIME2. Reads were then denoised into amplicon sequence variants (ASVs) and taxonomical classifications were assigned to ASVs using a pre-trained Naive Bayes classifier (41) from SILVA database (release 138) (42).

#### Prediction of α-1,3-Galactosyltransferase Genes in Penguin Microbiomes

We tested the presence and abundance of α1,3GT genes in the bacterial microbiomes of Humboldt penguins and, for comparative purposes, other selected penguin species. These include the Macaroni penguin (*E. chrysolophus*), the Little penguin (*E. minor*), the Gentoo penguin (*P. papua*), and the King penguin (*A. patagonicus*). The 16S rRNA amplicon sequences of Humboldt penguin (this study) and publicly available 16S rRNA datasets [for the other penguins, Bioproject PRJEB3083 (37)] were used to predict the metabolic profiling of each sample.

PICRUSt2 (43) was used to predict the metagenomes from 16S rRNA amplicon sequences. Briefly, the ASVs were placed into a reference tree (NSTI cut-off value of 2) containing 20,000 full 16S rRNA sequences from prokaryotic genomes, which was then used to predict individual gene family copy numbers for each ASV. The predictions are based on Kyoto Encyclopedia of Genes and Genomes (KEGG) orthologs (KO) (44). The analysis was focused in the detection of α1,3GT genes including the gspA-general secretion pathway protein A (accession K02450), waaL, rfaL-O-antigen ligase (K02847); waaO, rfaI-UDP-glucose: (glucosyl) LPS alpha-1,3-glucosyltransferase (K03275); waaJ, rfaJ, UDP-glucose: (galactosyl) LPS alpha-1,2-glucosyltransferase (K03279); waaR, waaT, rfaJ- UDP-glucose/galactose: (glucosyl) LPS alpha-1,2-glucosyl/galactosyltransferase (K03276) and waaI, rfaI-UDP-D-galactose: (glucosyl) LPS alpha-1,3-D-galactosyltransferase (K03278). The bacterial taxa contributing to these genes were also identified. A Sankey and chord diagrams were used to represent the taxa contribution analysis using the networkD3 (45) and circlize (46) packages, respectively, as implemented in R studio (47).

### Vaccination Trial

#### Penguins and Vaccination Trial Design

Before starting the vaccination trial, a preliminary safety test of the probiotic bacteria was used as a vaccine, and the placebo was carried out using two penguins (none of them included in the vaccination trial groups). One penguin was given an oral dose of vaccine and the other one a placebo. Both individuals were closely monitored by zookeepers for three weeks. Once the administration of the ‘oral vaccine’ and the placebo were considered safe for the penguins, the trial started.

Twenty adult Humboldt penguins, were randomly selected from the study colony (*n* = 123). Each penguin was assigned to either the vaccination (*n* = 10, penguins N°11-20) or the placebo (*n* = 10, penguins N°21-30) groups (**Table 1**). Penguins in each group were identified with wing tags of different colors. Based on medical records, clinical examination, and baseline plasma electrophoretic profiles, all animals included in the study were considered healthy at the moment of inclusion (See below details on ‘*diagnosis of aspergillosis*’). During the vaccination trial (vaccine and placebo given orally on D0, D2, D7, D9, D14, and D16; **Table 1**), and the follow-up period of one year, the animals were kept together with the other penguins of the colony.

**Table 1:** Penguins and procedures in the vaccination trial^a^.

Group	Penguin n°	Sex	Restraint sessions	Vaccine sessions						Restraint sessions	
			1^st^ Blood samplings #1 + CT #1 « T-1mo »	1^st^ Monday 21/12/20 PO	2^nd^ Wednesday 23/12/20 PO	3^rd^ Monday 28/12/20 PO	4^th^ Wednesday 30/12/20 PO	5^th^ Monday 04/01/21 PO	6^th^ Wednesday 06/01/21 PO	2^nd^ Blood samplings #2 « T1mo »	3^rd^ Blood samplings #3 + CT#2 « T3mo »
Vaccine group	11	F	17/12/2020	ok	ok	ok	ok	ok	ok	14/01/2021	31/03/2021
	12	F	17/12/2020	ok	2 doses	ok	ok	ok	ok	14/01/2021	31/03/2021
	13	F	17/12/2020	ok	ok	ok	ok	ok	ok	14/01/2021	31/03/2021
	14	M	16/12/2020^b^	ok	ok	ok	ok	ok	ok	14/01/2021	31/03/2021
	15	M	16/12/2020^b^	ok	ok	ok	ok	ok	ok	14/01/2021	31/03/2021
	16	M	16/12/2020^b,c^	ok	ok	ok	ok	ok	ok	14/01/2021	31/03/2021
	17	F	17/12/2020	ok	ok	ok	ok	ok	ok	14/01/2021	31/03/2021
	18	M	17/12/2020	ok	ok	ok	ok	ok	no	15/01/2021	31/03/2021
	19	M	17/12/2020	ok	ok	ok	ok	ok	ok	20/01/2021	31/03/2021^b,c^
	20	F	16/12/2020	ok	ok	ok	2 doses	ok	ok	14/01/2021	31/03/2021
Placebo group	21	F	16/12/2020	ok	ok	ok	ok	ok	ok	15/01/2021	31/03/2021
	22	M	16/12/2020^b^	ok	ok	ok	ok	ok	ok	14/01/2021	31/03/2021
	23	F	16/12/2020^b^	ok	ok	ok	ok	ok	ok	14/01/2021	31/03/2021
	24	M	16/12/2020	ok	ok	ok	ok + 1 dose of vaccine	ok	ok	14/01/2021	31/03/2021
	25	F	17/12/2020	ok	ok	ok	ok	ok	ok	14/01/2021	31/03/2021
	26	M	16/12/2020^b^	ok	ok	ok	ok	ok	ok	14/01/2021	31/03/2021
	27	F	17/12/2020	ok	ok	ok	ok	ok	ok	14/01/2021	31/03/2021
	28	F	20/12/2020^b^	ok	ok	ok	ok	ok	ok	14/01/2021	31/03/2021
	29	M	16/12/2020	ok	ok	ok	ok	ok	ok	14/01/2021	31/03/2021
	30	M	17/12/2020	ok	ok	ok	ok	ok	ok	14/01/2021	31/03/2021

a Two penguins from the vaccine group took two extra doses (penguins n°12 and n°20), while another one missed the last dose due to difficulties in animal handling (penguin n°18). These three individuals were kept into the vaccine group for statistical analyses. Penguin n°24 from the placebo group accidentally swallowed a dose of vaccine and was excluded from statistical analysis.

b Dorsal recumbency was needed, as excited penguins refused to stay straight in the PVC tube when standing.

c Flash isoflurane anesthesia (facemask) was performed, as these penguins were too fat to be placed appropriately into the PVC tube.

#### Oral Administration of Probiotic Bacteria and Placebo

Animals in the vaccination group received *E. coli* Nissle 1917 (Mutaflor, DSM 6601, Pharma-Zentrale GmbH, Herdecke, Germany). These probiotic bacteria were administered orally using gastro-resistant hard capsules (2.5 - 25 x 10^9^ colony-forming units (CFU) per capsule). Animals in the placebo group were given empty vegetable capsules made of pullulan (YourSupplements, Bredbury, Stockport, UK). The vaccine or the placebo capsules were orally administrated every Monday and Wednesday for three consecutive weeks (**Table 1**). Free-food sessions, in the large enclosure of the penguins at the zoo, were used to administer the vaccine or placebo capsules using thawed sprats (*Sprattus sprattus*) as baits.

#### Blood Sampling and Blood Processing

Three sampling sessions were performed one month before (T-1mo), one month (T1mo), and three months (T3mo) after the beginning of the vaccination protocol (**Table 1**). Penguins were manually restrained and 2-to-2.5ml of blood were sampled from either the medial metatarsal vein or the dorsal coccygeal vein (14), using a 2.5ml plastic syringe and a 22-g needle. For each penguin, 0.5ml of whole blood was immediately transferred to EDTA tubes (Tube BD^®^ Vacutainer^®^ Silicone EDTA 4ml), homogenized, then transferred to a dry tube containing 0.5ml of perchloric acid, homogenized again and stored at 4°C until further processing. Another 0.5ml was also immediately transferred to heparin-lithium tubes (Tube BD^®^ Vacutainer^®^ Silicone lithium heparin 4ml) and homogenized. The remaining amount of fresh blood (1-to-1.5ml) was transferred into a plastic tube for serum collection with a coagulation activator (Tube BD^®^ Vacutainer^®^ Silicone dry tube 4ml). All tubes were centrifuged at 5000rpm for 15 minutes twice. The supernatants obtained from the EDTA-blood + perchloric acid tubes were stored in cryotubes (Thermo Fischer Scientific, 91140 Les Ulis, France) at -20°C. Once the three sampling sessions were completed, samples were thawed and 3-hydroxybutyrate (3-OHB) concentration was measured using the Roche Cobas 6000^®^ instrument (Roche Diagnostics, Indianapolis, IN), with INstruchemie^®^ reagents (INstruchemie, Delfzijl, The Netherlands). Capillary zone electrophoretic profiles were obtained from the fresh heparin-lithium plasma samples using the Minicap^®^ automated system (Sebia, Lissex, 91008 Evry Cedex, France). For each plasma sample, total protein concentration was determined using a chemistry analyzer (Catalyst One, Idexx Laboratories, Inc., Westbrook, Maine 04092 USA). Serum was also extracted and stored at -20°C until further processing.

#### Computed Tomography

The twenty penguins of the trial were brought to the on-site veterinary hospital for computed tomography (CT) imaging (Philips Brilliance 64; Philips Medical Systems; Philips France, 92156 Suresnes, France; No iodine contrast. Acquisition parameters: 2 mm slice thickness, -1.5 mm increment, Tube voltage 120 kV, Tube current 150 mAs/slice; Pulmonary reconstruction parameters: 158mm FOV, 1mm slice thickness, -0.6mm increment, Y-detail (YB) filter) at two time points (T-1mo, T3mo, **Table 1**). Penguins were placed in a 40cm-high, 18cm-large PVC tube fixed on a rigid plate, as previously reported (48). If the penguins were too big to fit the PVC tube, they were anesthetized with isoflurane delivered by facemask to perform a dorsal recumbent CT scan. A binary scoring system was created to assess aspergillosis-related lesions. Six reading categories were considered: right lung nodules, left lung nodules, air sac nodules, air sac plaques, trachea nodules, and trachea plaques. For each category, the presence (1) or absence (0) of lesions was recorded for each penguin at T-1mo and T3mo. CT scans were blindly read (vaccination status not available), separately by two radiologists (European College of Veterinary Diagnostic Imaging diplomates). Both readings were harmonized to a collegiate, unique score for each CT scan. Size (mm) and the number of each lesion were also recorded.

#### Diagnosis of Aspergillosis

A multi-tool diagnostic panel (16, 17) was used to assess aspergillosis status at T-1mo, T1mo, and T3mo (**Table 1**). Criteria for suspicion of aspergillosis in penguins included clinical signs (increased respiratory efforts, abnormalities on respiratory auscultation), respiratory lesions on the CT scans (49, 50) (see the section above “Computed tomography”), 3-hydroxybutyrate plasma levels above the cut-off value of 940μmol/l together with an elevation of the α_2_ and β electrophoretic fractions (51), and elevated anti-*A. fumigatus* Abs (50, 52–54). Penguins were closely monitored by zookeepers each day, and any abnormal behavior was reported to the zoo veterinarians. If penguins from the study died during the one-year follow-up period (01/12/2020 – 01/12/2021), a complete *post-mortem* examination was performed together with a complete histopathology examination, as well as cytology, fungal culture, and *Aspergillus* sp. PCR on air sac and lung samples.

### Serological Assays and Analyses

#### Evaluation of Secondary Antibodies for Penguin Abs Detection

Considering the lack of commercially available anti-penguin Abs, we assessed the specificity of anti-chicken and anti-turkey Abs for penguin IgY for their use as secondary Abs in indirect ELISA assays to measure penguin Abs in sera samples. Specificity was assessed using indirect ELISA and western blots, as described below. For serological assay optimization, sera samples of different penguins (*n* = 10, **Supplementary Table S1**), not included in the vaccination trial, were used.

#### Indirect ELISA

Plates (Nunc-ImmunoTM Plate, Thermo Scientific, Waltham, MA, USA) were coated with α-Gal linked to Human Serum Albumin (HSA) (HSA-α-Gal, 50ng/well, Dextra Laboratories, Reading, UK) diluted in carbonate/bicarbonate buffer (0.05 M, pH 9.6) and incubated overnight at 4°C. Wells were washed three times with 100 µL of Phosphate-Buffered Saline: 10 mM NaH_2_PO_4_, 2.68 mM KCl, 140 mM NaCl, pH 7.2 (Sigma-Aldrich, St. Louis, MO, USA), (PBS) with 0.05% tween (PBST) and then blocked with 100 μL of 1% HSA/PBS for 1 hour at room temperature (RT). After three washes, sera samples, diluted 1:200 in PBS, were added to the wells and incubated for 1 hour at 37°C. The plates were washed three times and horseradish peroxidase (HRP)-conjugated goat anti-turkey IgY (Mybiosource, San Diego, USA) or goat anti-chicken IgY (Sigma-Aldrich, St. Louis, MO, USA) were added at dilution 1:1500 in PBS (100 μL/well) and incubated for 1 hour at RT. Subsequently, the plates were washed three times and the reaction was developed by adding 100 µL ready-to-use TMB solution (Promega, Madison, USA) at RT for 20 min in the dark. Then, 50 µL of 0.5 M H_2_SO_4_ was added to stop the reactions. The optical density (OD) was measured at 450 nm using a Multiskan FC ELISA reader (Thermo Scientific, Waltham, MA, USA). All samples were tested in triplicate and the average value of three blanks (no Abs) was subtracted from the reads. The cut-off was determined as two times a mean OD value of the blank controls.

#### Western Blots

Total chicken and penguin IgY were purified using the Pierce™ Chicken IgY Purification Kit kit (Thermo Scientific, Waltham, MA, USA), following the manufacturer’s instructions. Purified chicken or penguin IgY were loaded on a 4-15% Mini-protean TGX stain-free gel (Bio-Rad, Hercules, USA) and separated by SDS-electrophoresis 1 hour at 120V by duplicated. One of the gels was used for protein staining with Coomassie blue. The other gel was used for protein transference onto Trans-Blot Turbo Transfer Packs membrane (Bio-Rad, Hercules, USA). The membrane was blocked 2 hours with 1% HSA (Sigma-Aldrich, St. Louis, MO, USA)/PBS at RT, washed three times with PBST, and incubated at 4°C overnight with HRP-conjugated goat anti-turkey IgY (Mybiosource, San Diego, CA, USA), or goat anti-chicken IgY (Sigma-Aldrich, St. Louis, MO, USA) antibodies dilution 1:400 in PBS. Immunoreactive proteins were visualized with TMB solution (Promega, Madison, WI, USA).

### Relative Quantification of Penguin Abs

#### Indirect ELISA for Quantification of Penguin Anti-α-Gal IgM and IgY

The occurrence of anti-α-Gal IgY was measured in penguins not included in the vaccination trial (**Supplementary Table S1**). Anti-α-Gal IgM and IgY were also measured in the penguins included in the vaccination trial (*n* = 20) at different time points (T-1mo, T1mo, and T3mo) (**Table 1**). In both cases, IgY or IgM was measured by indirect ELISA, as described above with the following specifications. Penguin sera were diluted 1:200 in PBS. HSA-α-Gal was used as coating antigen and anti-chicken IgY or IgM (Sigma-Aldrich, St. Louis, MO, USA) were added at 1:1500 dilution in 1% HSA/PBS (100 μL/well). Reactions were developed and stopped as described above.

#### Competitive ELISA for Quantification of Total Penguin Anti-α-Gal Abs

Total penguin anti-α-Gal Abs were measured in animals included (**Table 1**), or not (**Supplementary Table S1**), in the vaccination trial. Total penguin anti-α-Gal Abs were measured as the capacity of penguin sera to inhibit the binding of the monoclonal mouse anti-α-Gal antibody (mAb) M86 (Enzo Life Sciences, Farmingdale, NY, USA) in a competitive ELISA (55), with modifications. Briefly, dilutions of penguin sera were prepared (1:200, 1:80, 1:20, and 1:5) and added to α-Gal antigen simultaneously with mAb M86. The binding of penguin sera to α-Gal was measured as mAb M86 binding inhibition percentage (%). Penguin sera were mixed with mAb M86 and both Abs were added simultaneously to plates coated with α-Gal-BSA (200 ng/well, Dextra Laboratories, Reading, UK). The HRP-conjugated goat anti-mouse IgM (Sigma-Aldrich, St. Louis, MO, USA) was used as a secondary antibody and the OD was measured at 450 nm with a Multiskan FC ELISA reader (Thermo Scientific, Waltham, MA, USA). Duplicates were used for each sample. The average value of the blanks was subtracted from all reads before further analysis. The mAb M86 binding inhibition % was calculated as {([OD mAb M86 alone] − [OD mAb M86 with penguin sera])/[OD mAb M86 alone]} × 100.

#### Relative Quantification of Penguin Anti-*A. fumigatus* IgM and IgY

Penguin Ab response to the fungus *A. fumigatus* was measured by indirect ELISA using protein extracts of *A. fumigatus* CBS 144.89 (CEA10) as coating antigens. *Aspergillus fumigatus* coating antigen was obtained by fungal culture and protein extraction. *Aspergillus fumigatus* culture was performed on Sabouraud dextrose agar (SDA), supplemented with chloramphenicol (5 mg/L), and incubated at 37°C for 10 days. Sub-cultures were performed twice a week and *A. fumigatus* colonies were grown for 2–3 days at 37°C. Conidia were subsequently harvested by resuspension in PBS with 0.01% tween, filtered in a 70 µm diameter nylon cell strainer (ClearLine Dominique Dutscher, Brumath, France), washed by centrifugation at 3500× g for 10 min, resuspended in with 0.01% tween and then counted using a Malassez counting chamber. *Aspergillus fumigatus* suspension containing 4 × 10^7^ conidia was resuspended in 500 µl lysis buffer PBS with 1% Triton All reagents used for the fungus preparation were apyrogenic. *Aspergillusfumigatus* proteins were extracted with six steel balls using the homogenizer Precellys^®^ 24 Dual (Bertin, France) at 5000× g for 20s, 2 times on lysis buffer. The homogenate was centrifuged at 200× g for 5 minutes and the supernatant was collected and quantified with BCA Protein Assay (Thermo Scientific, Waltham, MA, USA) with bovine serum albumin (BSA) as standard. Anti-*A. fumigatus* IgM and IgY were measured in penguin sera samples collected at T-1mo, T1mo, and T3mo (**Table 1**) by indirect ELISA, as described above with the following specifications. Penguin sera were diluted 1:200 in PBS. *Aspergillus fumigatus* protein extract was used as coating antigen and anti-chicken IgY or IgM (Sigma-Aldrich, St. Louis, MO, USA) were added at 1:1500 dilution in 1% HSA/PBS (100 μL/well). Reactions were developed and stopped as described above.

#### Relative Quantification of Penguin Anti-*E. coli* Nissle IgM and IgY

Penguin Ab response to the bacteria *E. coli* was measured by indirect ELISA using protein extracts of *E. coli* Nissle 1917 as coating antigens. *Escherichia coli* coating antigen was obtained by bacterial culture and protein extraction. *Escherichia coli* Nissle 1917 (Mutaflor strain) were grown on 20 mL of Luria Broth (Sigma-Aldrich, St. Louis, MO, USA), at 37°C overnight to OD_600_ = 0.8. Bacteria were washed twice with PBS, centrifuged at 1000× g for 5 min at 4°C, and resuspended in 500 µl lysis buffer, PBS with 1% Triton (Sigma-Aldrich, St. Louis, MO, USA). All reagents used for bacterial preparation were apyrogenic. *Escherichia coli* Nissle proteins were extracted with six steel balls using the homogenizer Precellys^®^24 Dual (Bertin, France) at 5000× g for 20s, 2 times on lysis buffer. The homogenate was centrifuged at 200× g for 5 minutes and the supernatant was collected and quantified with BCA Protein Assay (Thermo Scientific, Waltham, MA, USA) with BSA as standard. Anti-*E. coli* IgM and IgY were measured in penguin sera samples collected at T-1mo, T1mo, and T3mo (**Table 1**) by indirect ELISA, as described above with the following specifications. Penguin sera were diluted 1:200 in PBS. *Escherichia coli* Nissle protein extract was used as coating antigen and anti-chicken IgY or IgM (Sigma-Aldrich, St. Louis, MO, USA) were added at 1:1500 dilution in 1% HSA/PBS (100 μL/well). Reactions were developed and stopped as described above.

### Determination of α-Gal on *E. coli* by Flow Cytometry

The presence of the glycan α-Gal was measured in *E. coli* Nissle 1917 (Mutaflor strain) by flow cytometry as described (56). The strains *E. coli* O86:B7 (ATCC 12701) with high content of α-Gal and *E. coli* ClearColi*^®^* BL21 (DE3) (Lucigen, Middleton, Wisconsin, USA) with no LPS (where α-Gal is mainly present) were used as positive (26, 30, 56, 57) and negative (58) controls, respectively. The strain *E. coli* BL21 (DE3, Invitrogen, Carlsbad, CA, USA) with low α-Gal content (30, 56) was used for comparative purposes. All bacterial strains were grown as described above. After three washes in PBS, the bacteria were fixed for 30 minutes in 4% paraformaldehyde, and then incubated with mAb M86 (Enzo Life Sciences, Farmingdale, NY, USA) diluted 1:100 in PBS. FITC-goat anti-mouse IgM (Sigma-Aldrich, St. Louis, MO, USA) labelled antibody (diluted 1:1000 in PBS 2 h at RT) was used as a secondary antibody. The mean fluorescence intensity (MFI) was determined by flow cytometry and compared between test and control cells.

### Purification and Quantification of Total IgY From Humboldt Penguins Egg Yolk

Two Humboldt penguin eggs were kindly provided by Sylvie Laidebeure and Alexis Lécu from the Paris Zoological Park (Muséum national d’Histoire naturelle, France). Egg yolk and egg white were separated. Total egg IgY was purified with Pierce™ Chicken IgY Purification Kit (Thermo Scientific, Waltham, MA, USA) following the manufacturer’s instructions. Briefly, egg yolk was weighed and mixed with a volume of cold delipidation reagent equal to five times the egg yolk weight. The mix was incubated for 2 hours at 4°C and then centrifuged 20 minutes at 10,000× g in a refrigerated centrifuge. The supernatant was recovered and an equal volume of cold IgY precipitation reagent was added and incubated for 2 hours at RT. After 20 minutes of centrifugation at 10,000× g in a refrigerated centrifuge, the pellet was collected and a volume of PBS equal to the original volume of the egg yolk was added. Total proteins were quantified using BCA Protein Assay (Thermo Scientific, Waltham, MA, USA) with BSA as standard. Egg Abs were quantified by competitive (total anti-α-Gal Abs) and indirect (anti-α-Gal, anti-*E. coli*, and anti-*A. fumigatus* IgY) ELISA, as described above.

### Statistical Analyses

Statistical differences of parameters measured in penguins of each group were evaluated using paired and unpaired Student’s *t*-test applied to individual paired and unpaired values, respectively. Pearson correlation was used to assess the correlation between anti-α-Gal and anti-*E. coli* Abs levels. All statistical analyses were performed in the GraphPad 9 Prism program (GraphPad Software Inc., San Diego, CA, USA). Differences were considered significant when *p* < 0.05.

## Results

### Presence and Distribution of α1,3GT Genes in Penguin Microbiomes

**Figure 1:**
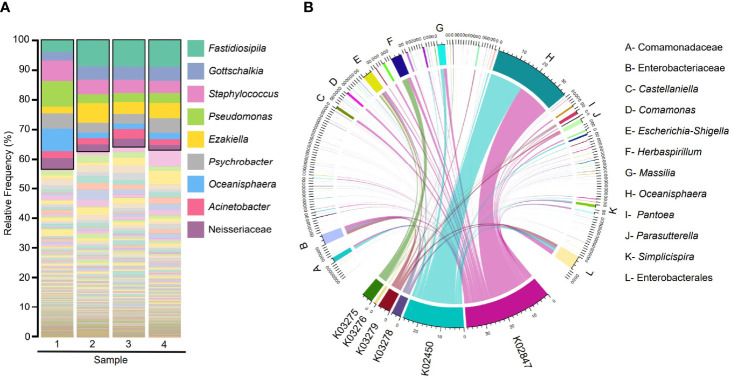
Taxonomic and functional profiles of the fecal microbiome of Humboldt penguin. **(A)** Barplot showing the taxonomic classification of gut bacterial species from Humboldt penguin. The nine taxa with the highest relative abundance were highlighted within the black box and shown in the legend. **(B)** Chord diagrams representing the metagenome contribution to α1,3GT genes in the gut microbiota of Humboldt penguin. The links indicate the linkage between the taxa and α1,3GT genes and their width are proportional to the contribution. Node segments along the circle represent taxa or functional genes, the node’s width represent the abundance (measured as feature count) of contributing taxa and genes. Only taxa with contribution higher than 1% to all the functional genes were shown in the legend.

Taxonomic classification of the microbiota of Humboldt penguin analyzed from fecal samples showed that the most abundant taxa were the genera *Fastidiosipila, Gottschalkia, Staphylococcus, Pseudomonas, Ezakiella, Psychrobacter, Oceanisphaera, Acinetobacter*, and Neisseriaceae (**Figure 1A**). The taxa Enterobacteriaceae and *Escherichia-Shigella* were found in all the samples, although their relative abundances were not high (less than 2%) (**Supplementary Table S2**). Furthermore, α1,3GT genes were predicted from the gut microbiome of Humboldt penguin and the taxa contributing to these genes were traced (**Figure 1B**). Results showed that 198 taxa contributed to the six α1,3GT genes but the contribution of only 12 of these taxa (listed in **Figure 1B**) was higher than 1%. Interestingly, among the taxa with the highest contribution, Enterobacteriaceae, and *Escherichia-Shigella* were found.

In addition to *S. humboldti*, the presence of α1,3GT genes was also predicted in the gut microbiome of other penguin species including *E. chrysolophus*, *E. minor*, *P. papua*, and *A. patagonicus*. More than twenty bacterial taxa, with a contribution higher than 1%, were identified to harbor α1,3GT genes (**Supplementary Figure S1**). Not all taxa contributed equally to the distribution of α1,3GT genes, as various of these taxa had very low abundance in the microbiomes and thus contributed only marginally to α1,3GT genes. Similar to the Humboldt penguin, all the α1,3GT genes were present in the microbiome of other penguin species studied. When taxa with the marginal contribution to the α1,3GT genes (less than 1%) were excluded, only four of the genes were found (i.e., K02450, K02847, K03278, K03279) to have a major presence in *E. chrysolophus*, *E. minor*, and *A. patagonicus* (**Supplementary Figure S1**). The most frequently found α1,3GT genes were K02450 and K02847, especially in *E. minor*. Among the four species analyzed, *E. minor* is the closest relative to the Humboldt penguin. Accordingly, Humboldt penguins presented a similar α1,3GT genes profile to *E. minor*, where K02450 and K02847 were also the most frequently found (**Figure 1B**). The taxa that most contributed to α1,3GT genes in *S. humboldti* were *Oceanisphaera.* In the other penguin species, the bacterial taxa *Gallicola*, PeH15, *Fusobacterium*, and Peptostreptococcales-Tissierellales were among the main contributors to α1,3GT genes. These results suggest a wide distribution of α1,3GT genes in the penguin microbiome, which may be associated with the induction of natural anti-α-Gal Abs in this group of birds.

### Occurrence of Anti-α-Gal Ab in Penguin Sera and Eggs

Western blot analysis showed that anti-chicken IgY recognizes bands of similar size in chicken and penguin IgY (**Supplementary Figure S2**). Anti-turkey IgY recognized several bands in purified chicken and penguin IgY (**Supplementary Figure S2**). In agreement with a non-specific recognition of anti-turkey IgY for penguin Abs, the indirect ELISA showed that the OD values using the anti-turkey IgY were higher than those using the anti-chicken IgY (data not shown). Based on the western blot and indirect ELISA results, anti-chicken secondary Abs were used in the indirect ELISA assays to measure the levels of penguin IgM and IgY.

**Figure 2:**
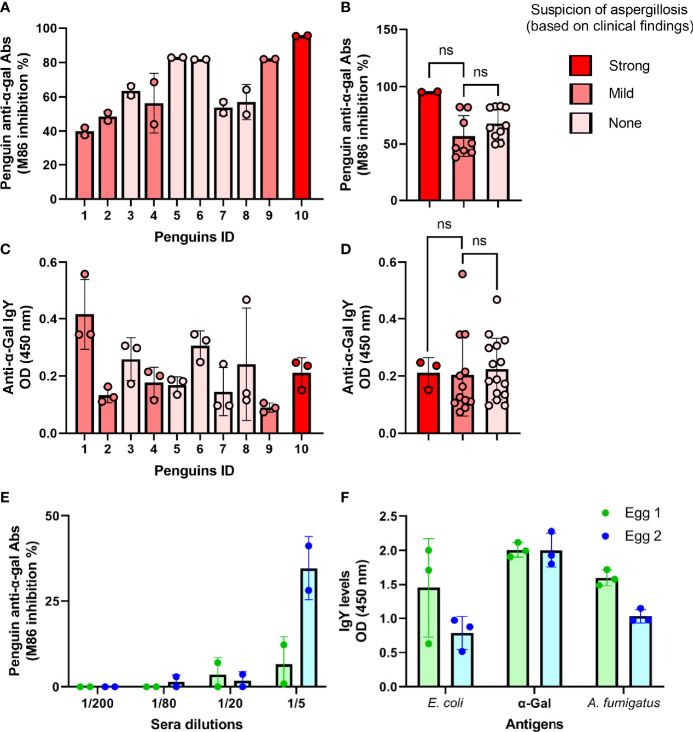
Individual variation of anti-α-Gal Abs in penguin sera and eggs. The levels of circulating total anti-α-Gal Ab **(A, B)** and anti-α-Gal IgY **(C, D)** were measured by competitive and indirect ELISA, respectively. Total and IgY anti-α-Gal Abs were variable in the sera of individual penguins (ID 1 - 10). Total anti-α-Gal Ab **(C)** and anti-α-Gal IgY **(D)** were measured in two egg yolks by competitive and indirect ELISA, respectively. Egg anti-α-Gal IgY specific to *E coli* and *A. fumigatus* proteins were measured by indirect ELISA. M86 binding inhibition % **(A, B, E)** and OD **(C, D, F)** means and standard deviation values of technical replicates *per* sample are displayed. Penguins with strong (Red), mild (rose) or none (light rose) clinical suspicion of aspergillosis were included, as listed in **Supplementary Table S1**. Values were compared by unpaired **(B, D)** student’s *t*-test (ns, not significant, strong (*n* = 1), mild (*n* = 4) and none (*n* = 5), and three technical replicates per sample).

The occurrence of anti-α-Gal Abs in Humboldt penguins was assessed in sera samples collected from healthy penguins (*n* = 5) and penguins with clinical suspicion of aspergillosis (*n* = 5) (**Supplementary Table S1**). At the individual level, total anti-α-Gal Abs were higher in the serum of the penguin with a strong suspicion of aspergillosis compared with that of the healthy animals (**Figure 2A**), but statistical analysis showed no difference between groups (*p* > 0.05, **Figure 2B**). Isotype-specific detection of penguin anti-α-Gal showed variable levels of IgY in this group of animals (**Figure 2C**), but differences were not significant (*p* > 0.05, **Figure 2D**). Total anti-α-Gal Abs (**Figure 2E**) and anti-α-Gal IgY (**Figure 2F**) were also detected in Humboldt penguin eggs. Antibodies IgY specific to *E. coli* and the pathogen *A. fumigatus* were also detected in the eggs (**Figure 2F**).

### Presence of α-Gal Glycan in the Probiotic *E. coli* Nissle

The α-Gal glycan was detected on the surface of *E. coli* Nissle as assessed by flow cytometry (**Table 2**). The α-Gal glycan was also detected in the positive control *E. coli* O86:B7. This was not the case for *E. coli* ‘clean’, and *E. coli* BL21 strains, used as negative controls of α-Gal production (**Table 2**). The average fluorescein isothiocyanate (FITC) absorption values were higher for *E. coli* Nissle and *E. coli* O86:B7 compared to those of *E. coli* ‘clean’ and BL21 strains (**Table 2**).

**Table 2:** Presence of α-Gal in *E. coli* Nissle measured by flow cytometry.

*E. coli* strains	FITC (AVG ± SD)	Comment
*E. coli* Nissle	1536 ± 9417	Test strain
	143 ± 130	Negative control for *E. coli* Nissle*
*E. coli* O86:B7	634 ± 5374	Positive control of α-Gal production
	123 ± 17	Negative control for *E. coli* O86:B7*
*E. coli* clean	218 ± 839	Negative control for α-Gal production.
	200 ± 581	Negative control for E. coli clean*
*E. coli* BL21	128 ± 47	Negative control for α-Gal production.
	124 ± 38	Negative control for *E. coli* BL21*

*FITC background value resulting from the secondary antibody alone.

### Modulation of Anti-α-Gal Abs After Oral Administration of *E. coli* Nissle

Before starting the trial, two penguins from the colony (not included in the trial) were randomly selected and given an oral dose of vaccine or placebo. No adverse effects were reported on these two individuals during three weeks of close monitoring. Based on these observations the vaccination trial started and oral administration of vaccine or placebo followed the schedule described in **Table 1**. No side effects were noted in any of the penguins administered the vaccine or the placebo.

**Figure 3:**
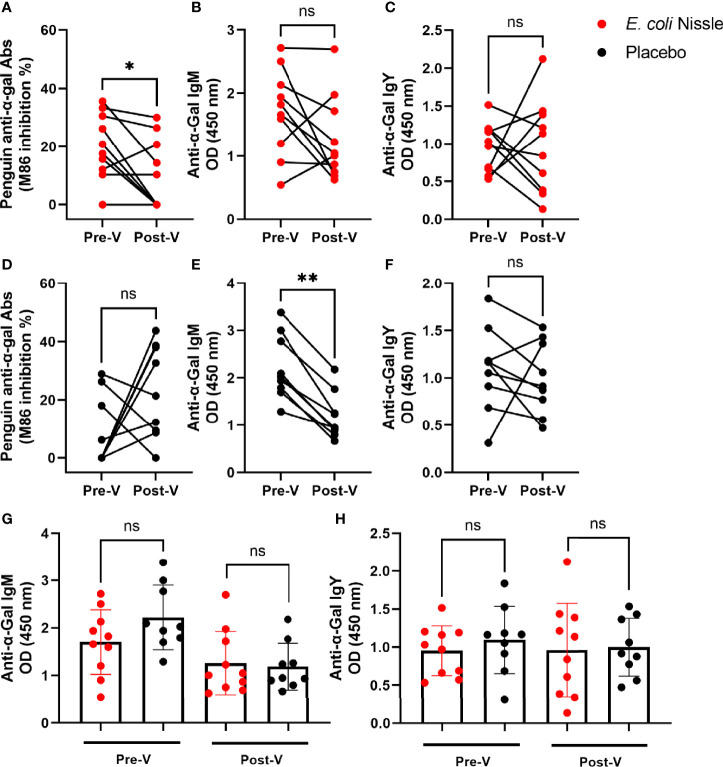
Variation in penguin anti-α-Gal Ab levels after oral administration of *E coli* Nissle. The levels of circulating total anti-α-Gal Ab **(A)** and anti-α-Gal IgM **(B)** and IgY **(C)** were measured by competitive (A, D) and indirect **(B, C**, **E, F**–**H)** ELISA in the *E coli* Nissle (red dots) and placebo (black dots) groups before (Pre-V) and after (Post-V) the vaccination protocol. Total anti-α-Gal Abs decreased in the sera of penguins vaccinated with oral *E coli* Nissle **(A)**. Anti-α-Gal IgM decreased in the sera of penguins of the placebo group **(E)**. No significant differences were observed in the levels of anti-α-Gal IgM **(G)** and IgY **(H)** when the two groups, *E coli* Nissle and placebo, were compared Pre-V and Post-V. Individual **(A–F)** and means and standard deviation **(G, H)** values are shown. Values were compared by paired **(A–F)** or unpaired **(G, H)** student’s *t*-test (*p < 0.05, **p < 0.005; ns, not significant, 1 experiment, n = 10 in the *E coli* Nissle group and n = 9 in the placebo group, and three technical replicates per sample).

Three months (T3mo) after the oral vaccination (Post-V), the penguins that received *E. coli* Nissle showed a significant reduction (10.19 SD ± 12.05, 95% IC 1.57-18.81) in total anti-α-Gal Abs compared with the levels before the oral vaccination (Pre-V, 20.20 SD ± 11.31, 95% IC 12.11-28.29) (Student’s *t*-test *p* = 0.03, **Figure 3A**). No differences were observed in the levels of anti-α-Gal IgM (**Figure 3B**) and IgY (**Figure 3C**) Post-V compared with the levels Pre-V. The animals in the placebo group showed no significant difference in the levels of total anti-α-Gal Abs (**Figure 3D**) and anti-α-Gal IgY (**Figure 3F**) before and after administering the placebo, while in the same group of animals, the levels of anti-α-Gal IgM were significantly lower Post-V (0.66 SD ± 0.49, 95% IC 0.80-1.56) compared with the levels Pre-V (2.22 SD ± 0.68, 95% IC 1.69-2.74) (Student’s *t*-test *p* = 0.003, **Figure 3E**). No significant differences were found in the levels of anti-α-Gal IgM (**Figure 3G**), or anti-α-Gal IgY (**Figure 3H**) Pre-V or Post-V in the vaccination compared with the placebo group.

**Figure 4:**
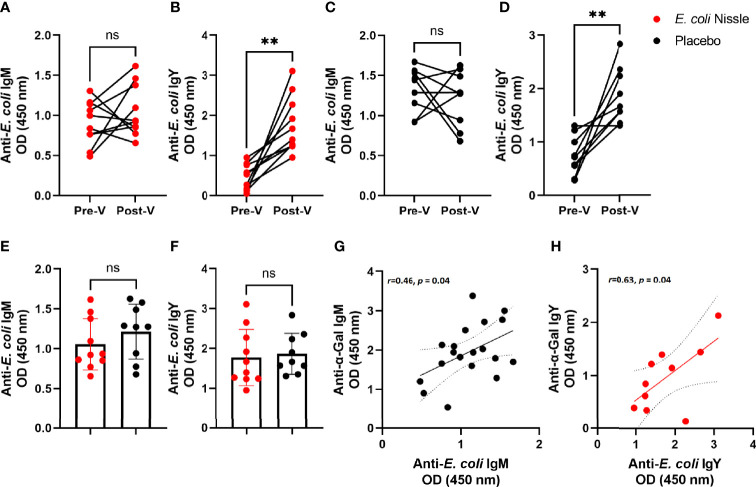
Levels of anti-*E. coli* Abs after oral administration of *E. coli* Nissle and their association with anti-α-Gal Ab levels. The levels of anti-*E. coli* IgM **(A, C, E)** and IgY **(B, D, F)** were measured in penguin sera by indirect ELISA in animals of the *E. coli* Nissle (red dots) and placebo (black dots) groups before (Pre-V) and after (Post-V) the vaccination protocol. No significant differences were observed in the levels of anti-*E. coli* IgM within **(A, C)** and between **(E)** groups. Significant differences were observed in the levels of anti-*E. coli* IgM within **(A, C)**, but not between **(E)** groups. Positive correlations between the levels of anti-*E. coli* IgM and anti-α-Gal IgM Pre-V (Pearson coefficient *r*=0.46, *p* = 0.04) **(G)**, and anti-*E. coli* IgY and anti-α-Gal IgY in the *E. coli* Nissle group Post-V (Pearson coefficient *r*=0.63, *p* = 0.04) **(H)** were found. Individual **(A–H)** and means and standard deviation **(E, F)** values are shown. Values were compared by paired **(A–D)** or unpaired **(E, F)** student’s *t*-test (**p < 0.005; ns, not significant, 1 experiment, n = 10 in the *E. coli* Nissle group and n = 9 in the placebo group, and three technical replicates per sample, A-F). Values of both groups placebo and *E. coli* Nissle were merged for the Pre-V anti-*E. coli*-anti-α-Gal IgM correlation analysis (n = 19). For the correlation of anti-*E. coli*-anti-α-Gal *E. coli* Nissle Post-V IgY n = 10.

We then asked whether oral administration of *E. coli* Nissle elicits anti-*E. coli* Abs and, if yes, whether a correlation existed between anti-*E. coli* and anti-α-Gal Abs levels. No significant change was found in the levels of anti-*E. coli* IgM Pre-V and Post-V (**Figure 4A**), while anti-*E. coli* IgY (**Figure 4B**) showed a significant increase in the penguins of the *E. coli* Nissle group. However, the same pattern was found in the placebo group, with no significant change in anti-*E. coli* IgM Pre-V and Post-V (**Figure 4C**) and a significant increase in anti-*E. coli* IgY (**Figure 4D**). The levels of anti-*E. coli* IgM (**Figure 4E**) and anti-*E. coli* IgY (**Figure 4F**) in the penguins of the *E. coli* Nissle group were not different from those of the placebo group. Positive correlations between the levels of anti-*E. coli* IgM and anti-α-Gal IgM Pre-V (Pearson coefficient *r*=0.46, *p* = 0.04, **Figure 4G**) and anti-*E. coli* IgY and anti-α-Gal IgY in the *E. coli* Nissle group Post-V (Pearson coefficient *r*=0.63, *p* = 0.04, **Figure 4H**) were found. There was no correlation between levels of anti-*E. coli* IgM or IgY and anti-α-Gal IgM or IgY in the placebo group Post-V or between anti-*E. coli* IgM and anti-α-Gal IgM in the *E. coli* Nissle group Post-V.

### Markers of *Aspergillus* Infection in Penguin Plasma

**Figure 5:**
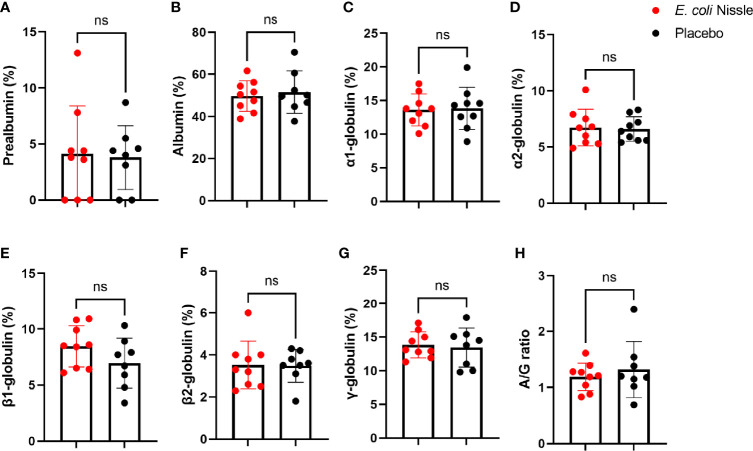
Levels of plasma proteins after oral administration of *E coli* Nissle. The percentage of prealbumin **(A)**, albumin **(B)**, α1 **(C)**, α2 **(D)**, β1 **(E)**, β2 **(F)**, and γ **(G)** -globulins concentrations and the albumin/globulin **(H)** ratio of animals in the *E coli* Nissle (red dots) and placebo (black dots) groups Post-V are shown. Individual, means and standard deviation values are shown. Values were compared by unpaired student’s *t*-test (ns, not significant, 1 experiment, n = 9 in the *E coli* Nissle group and n = 8 in the placebo group).

Oral administration of *E. coli* Nissle was not associated with significant changes in the prealbumin, albumin, α1, α2, β1, β2, and γ-globulins concentrations and albumin/globulin (A/G) ratio compared with the placebo group (**Figure 5**). Except for prealbumin, the levels of the above plasma proteins did not change significantly Post-V in the *E. coli* Nissle or placebo groups compared with the levels Pre-V (**Supplementary Figure S3**). In the placebo group, the levels of prealbumin were significantly lower Post-V (3.36 SD ± 2.95, 95% IC 1.09-5.64) compared with the levels Pre-V (6.56 SD ± 1.37, 95% IC 5.50-7.62) (Student’s *t*-test *p* = 0.01), but the level of this plasma protein did not change significantly in the *E. coli* Nissle group (**Supplementary Figure S3**).

**Figure 6:**
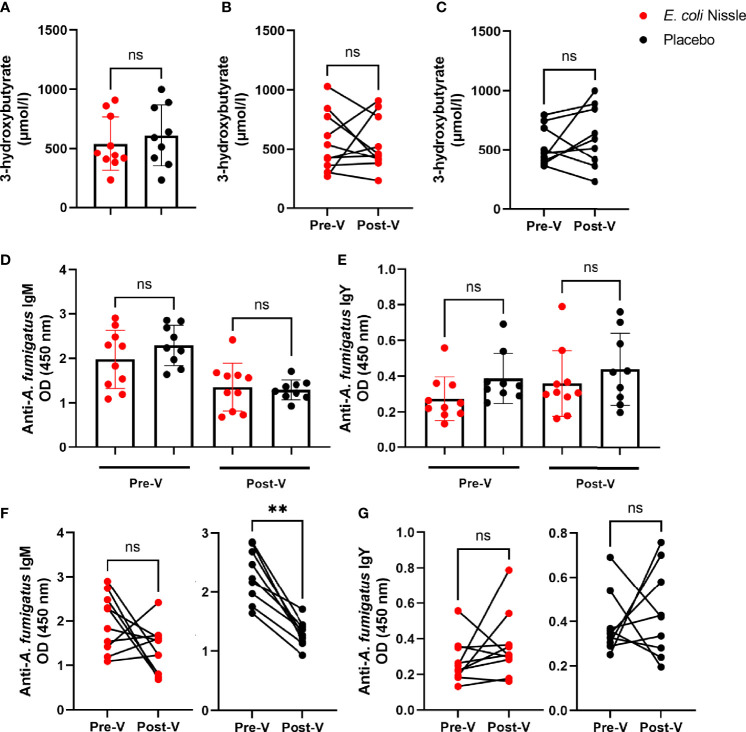
3-OHB plasma levels and anti-*A. fumigatus* Abs after oral administration of *E coli* Nissle. The concentration of 3-OHB (µmol/L) in the *E coli* Nissle (red dots) and placebo (black dots) groups were compared between groups **(A)** and within *E coli* Nissle **(B)** or placebo **(C)** groups before (Pre-V) and after (Post-V) the vaccination protocol. Anti-*A. fumigatus* IgM **(D, F)** and IgY **(E, G)** levels in penguin sera were measured by indirect ELISA and compared between **(D, E)** and within **(F, G)** groups. Individual **(A–G)** and means and standard deviation **(A, D, E)** values are shown. Values were compared by paired **(B, C, F, G)** or unpaired **(A, D, E)** student’s *t*-test (**p < 0.005; ns, not significant, 1 experiment, n = 10 in the *E coli* Nissle group and n = 9 in the placebo group, and three technical replicates per sample in the ELISA).

The mean concentration of 3-OHB Post-V in penguins of the *E. coli* Nissle group was lower (540.3 SD ± 226.9 µmol/L) than that in the placebo group (610 SD ± 259.1 µmol/L), but the difference was not statistically significant (Student’s *t*-test *P* > 0.05, **Figure 6A**). The levels of 3-OHB before and after the oral vaccination did not change significantly in the penguins of *E. coli* Nissle (**Figure 6B**) or placebo groups (**Figure 6C**). Individual values of 3-OHB per penguin are available in **Supplementary Table S3**.

The levels of IgM (**Figure 6D**) and IgY (**Figure 6E**) specific to *A. fumigatus* were not significantly different between groups (*E. coli* Nissle *vs*. placebo) Pre-V or Post-V. Oral administration of *E. coli* Nissle was not associated with significant changes in anti-*A. fumigatus* IgM levels before and after the treatment, while the levels of IgM (2.29 SD ± 0.45, 95% IC 1.94-2.63) were significantly lower Post-V (1.29 SD ± 0.22, 95% IC 1.12-1.46) in the penguins of the placebo group (Student’s *t*-test *p* = 0.0001, **Figure 6F**). No significant changes were observed in the levels of anti-*A. fumigatus* IgY Pre-V and Post-V in the *E. coli* Nissle or placebo groups (**Figure 6G**).

### Morbidity and Mortality Occurrence in Vaccinated vs. Non-Vaccinated Penguins

**Figure 7:**
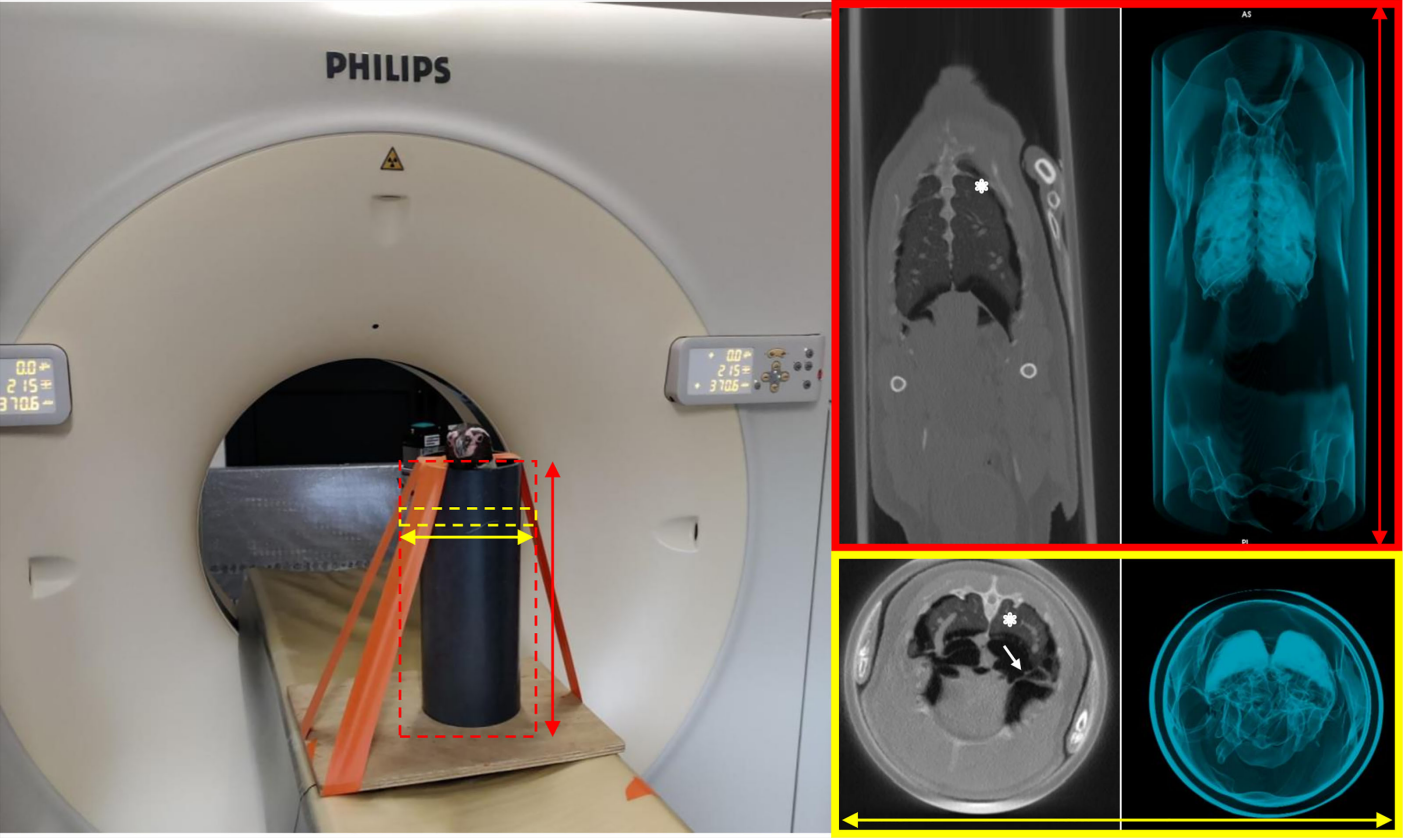
Computed tomography in a non-anesthetized, standing Humboldt penguin. Dorsal (top right) and transverse (bottom right) sections of penguin n°13 are presented. The lung parenchyma (asterisks) and the thoracic air sacs walls (arrow) are well depicted. No lesions (nodules or plaques) are seen.

During the first session, 13 penguins underwent conscious, standing scanning using a device inspired by Rivas et al. (48) (**Figure 7**), while the remaining seven underwent conscious CT scanning in dorsal recumbency. No pulmonary lesion was observed in any of the penguins at any time point (T-1mo and T3mo), considering any of the six reading categories (**Supplementary Table S3**).

Three penguins exhibited respiratory signs consistent with aspergillosis after the trial, one from the vaccine group and two from the placebo group. A 7.5-yr-old, vaccinated male (penguin n°14) died from respiratory aspergillosis eight months after the trial (*A. fumigatus* isolated from extensive lesions in the left caudal air sac and the lungs, by PCR and fungal culture), despite antibiotic and antifungal treatments. Strong suspicion of respiratory aspergillosis occurred in two females from the placebo group (penguin n°23 three days after the second blood sampling session, and penguin n°25 eight months after the trial), whose clinical signs (lethargy, dysorexia, intermittent increased respiratory noises) resolved after the completion of antibiotic and antifungal treatments for ten to 21 days. They were considered healed based on normal behavior, appetite, physical examination, and follow-up blood work.

## Discussion

Aspergillosis is a major cause of morbidity and mortality in captive-bred penguins (4–6) and treatment success is often hampered by diagnostic difficulty and few identifiable clinical signs. Preventive measures are paramount in zoological institutions housing penguins, and limitation of risk factors (stress, poor nest sanitation, poor ventilation, high temperature and humidity, high number of individuals living together, respiratory airways irritation by aerosolized toxins, and others) is essential to avoid the development of the fungal infection (3, 6, 7). The development of a vaccine makes it possible to consider long-term protection, but previously published studies do not show the efficacy of the tested strategies (13, 59, 60), in penguins or other birds.

The presence of α-Gal on the surface of *A. fumigatus* (30) may explain variations of anti-α-Gal Abs in *A. fumigatus*-infected birds. Variations in anti-α-Gal Abs levels in healthy individuals have been associated with the bacterial composition of the gut microbiome in humans (35, 61), α1,3GT-deficient mice (62), and fish (33). Production of anti-α-Gal Abs in humans is thought to be driven by intestinal exposure to bacteria of the *Klebsiella*, *Serratia*, and *Escherichia* genera expressing α-Gal (61). In addition to these bacterial genera, α1,3-GT genes are broadly distributed in the bacterial gut microbiome of humans (63), and wild and domestic birds (64). In this study, some of these bacterial taxa were found to contribute to the pool of α1,3-GT genes in the microbiome of the penguins *E. chrysolophus, E. minor, P. papua, A. patagonicus*, and *S. humboldti*. Individual differences in the gut microbiome of *S. humboldti* could explain the presence of anti-α-Gal Abs in healthy penguins. The positive correlation between the levels of anti-α-Gal and anti-*E. coli* IgM before the treatment further suggests a role of penguin gut microbiota in the production of anti-α-Gal Abs. Several studies show that the inter-generational transfer of maternal Abs provides humoral immune defence against pathogens in eggs and early-life offspring. This mechanism is crucial as endogenous production of Abs in chicks occurs only 10-14 days post-hatching (65). Anti-*Aspergillus* Abs may be transmitted from mother to offspring in penguins (3), a finding confirmed by the presence of anti-*Aspergillus* IgY in the two Humboldt penguin eggs tested in our study. Our results also showed the presence of anti-α-Gal Abs in Humboldt penguin eggs. The levels of anti-α-Gal IgY in eggs of different bird species are variable (65–67). Anti-α-Gal IgY isolated from birds can bind α-Gal antigens in mammalian tissues. Particularly, the binding of avian anti-α-Gal Abs blocks the binding of human anti-α-Gal to xenograft endothelial cells (68, 69). Avian anti-α-Gal Abs also block human blood complement activation and antibody-dependent cell-mediated lysis mechanisms that are responsible for hyperacute rejections in xenografts (68, 69). This shows the functionality of avian anti-α-Gal Abs. However, whether anti-*Aspergillus* and/or anti-α-Gal Abs inter-generationally transferred from the mother to egg to chick, have protective functions against *Aspergillus* or other infectious diseases in penguins remains an open question.

The role of microbiota in the induction of anti-α-Gal Abs has been experimentally evaluated. Gut colonization by *E. coli* O86:B7 elicited anti-α-Gal Abs in α1,3GT-deficient mice (57), humans (70), primates (71), white Leghorn chicks (72), and turkeys (30). Modulation of anti-α-Gal immunity using gut microbiota manipulation protects birds against avian aspergillosis, caused by experimental infection with *A. fumigatus* (30). Specifically, oral administration of *E. coli* O86:B7 increased the levels of IgY against the disaccharide Galα1-3Gal in sera of treated turkeys. Oral administration of *E. coli* O86:B7 was also associated with decreased anti-α-Gal IgA in lungs compared with non-treated turkeys. Decreased levels of anti-α-Gal IgA were accompanied by a reduction in the occurrence of lung granulomas, which is associated with acute aspergillosis in turkeys. These results suggest a crosstalk mechanism in birds by which the gut microbiota modulates the immune response in the lungs (73). In the infection model reported by Mateos-Hernández et al., 2020 (30), (i.e., the intratracheal infectious challenge with *A. fumigatus*), the mechanism of protection against avian aspergillosis does not seem mediated by increased anti-Galα1-3Gal IgY in sera. However, increased sera levels of anti-Galα1-3Gal IgY induced by oral administration of *E. coli* O86:B7, or other *E. coli* strain producing α-Gal may be relevant to prevent avian aspergillosis in conditions of airborne infection with *A. fumigatus*, as occur in natural conditions. Here we showed that *E. coli* Nissle produces α-Gal levels similar to those found in *E. coli* O86:B7 and higher than those found in α-Gal-negative bacteria, as measured by flow cytometry. In the present study, oral administration of *E. coli* Nissle was not associated with significant changes in the levels of anti-α-Gal IgY in penguins. However, we found a significant decrease in total anti-α-Gal Abs of animals treated with *E. coli* Nissle. Interestingly, oral administration of *E. coli* Nissle in penguins prevented the sharp decrease in anti-α-Gal IgM observed in the placebo group. Possible explanations for these differences are the species (turkeys *vs.* penguins), the bacterial dose (three administrations *per* week for three weeks in turkeys (30) *vs.* two administrations *per* week for three weeks in penguins), or a differential cellular location of the α-Gal epitope in *E. coli* Nissle and *E. coli* O86:B7. The α-Gal epitope of *E. coli* O86:B7 is expressed on the capsule or glycoprotein portion of the bacterial wall, rather than on the LPS molecules (61). Other *E. coli* strains were found to express the glycan in the LPS molecules (61). These findings suggest that the α-Gal epitope can be located in the LPS, capsule, or glycoprotein portion of *E. coli*. The cellular fraction in which *E. coli* Nissle expresses α-Gal is currently unknown. The results of the present study suggest that *E. coli* Nissle express α-Gal in a cellular fraction that is not accessible to the immune system to induce a significant increase of anti-α-Gal Abs. However, the positive correlation between the levels of anti-α-Gal and anti-*E. coli* IgY in the *E. coli* Nissle group suggests that the immune response to α-Gal is associated with that triggered by *E. coli*.

For aspergillosis and diagnostic tools in penguins, CT is a state-of-the-art diagnostic method for respiratory symptoms in penguins (74, 75) and was therefore included in the diagnostic panel for aspergillosis assessment in the study penguins. Physiological alterations in the lung (density and volume) and air-sac (volume) readings have been associated with dorsal recumbency (75) in Humboldt penguins undergoing CT, while no difference was seen in conscious *vs.* anesthetized individuals. Thus, the decision was made to perform CT in non-anesthetized penguins in a standing positioning as far as possible (**Figure 7**), as previously described (48). Other diagnostic tools included physical examination, plasma levels of 3-hydroxybutyrate, capillary zone electrophoresis, and *A. fumigatus* serology. A recently described method using an *Aspergillus* lateral-flow device is promising when combining plasma and glottis mucus swab samples but was not included in our study (76). Three penguins exhibited clinical signs consistent with respiratory aspergillosis. Penguin n°14 died from disseminated aspergillosis and penguin n°25 was successfully treated on strong clinical suspicion. As both of them presented seven to eight months after the vaccination trial, these findings were considered unrelated to the trial. Penguin n°23 was also successfully treated after a strong suspicion of respiratory aspergillosis reported three days after the second blood sampling session (T1mo), which was considered the stressful event that led to acute immunosuppression and subsequent acute form of aspergillosis.

Definitive diagnosis of aspergillosis relies on definitive fungal identification (by fungal culture or PCR) and typical morphological and histological lesions. *Ante-mortem* diagnosis remains difficult, and a multi-tools analysis is required (2, 16, 77). Plasma electrophoresis offers valuable data in infected penguins (51, 78–80) especially the β globulin fraction containing the fibrinogen (53, 77, 80–82), and 3-OHB levels (83). For these reasons, capillary zone electrophoresis assay and measurement of 3-OHB levels were added to the diagnostic panel in this study. Despite being a sometimes-useful assay in selected avian species such as psittacine birds (53, 80, 84) the s=galactomannan assay is not reliable in penguins (85) and was not used in the study.

Three months after the beginning of the trial, vaccinated penguins showed lower plasmatic levels of 3-OHB compared to placebo penguins, but no statistical difference was seen. Whether the lack of significance is due to insufficient statistical power and the oral vaccine is associated with a slower progression of subclinical *Aspergillus* sp. infection as reflected by lower 3-OHB values would be overstated. Mean values were below previously reported values (51), in healthy African penguins (*Spheniscus demersus*), a species closely related to the Humboldt penguin. For accurate aspergillosis assessment in penguins, plasma levels of 3-OHB should be interpreted together with β-globulins and α2 globulins (51) in order to have a high specificity (> 90%) and a good negative predictive value (≥ 80%). No significant difference was seen in these electrophoretic fractions between penguin groups. These results suggest that none of the penguins had developed aspergillosis during the three months of the trial, which is consistent with CT scan readings throughout the study period. As a consequence, differences observed between penguin groups in Abs levels Post-V were only attributable to the oral vaccine compared to the placebo.

However, a significant reduction of the pre-albumin fraction was observed on the electrophoretic profiles of non-vaccinated penguins after the vaccination trial. This reduction was not seen in the vaccination group. This finding suggests that prealbumin concentrations decrease when globulin levels rise (86). The only relevant difference in Abs levels Pre-V *vs*. Post-V was a significant decrease of total anti-α-Gal Abs in vaccinated penguins and of anti-α-Gal IgM in non-vaccinated individuals. Consequently, a direct link between globulin levels and prealbumin concentrations seems highly unlikely in our study population and the exact clinicopathological relevance of this finding remains unknown. Still, a link with non-humoral immunity pathways is hypothesized. Significantly lower prealbumin values have been reported in falcons with confirmed aspergillosis (77, 82). In humans, a similar trend was seen in fatal cases of COVID-19 (87). As a transport protein, the human prealbumin fraction (also so-called transthyretin) has various physiological effects, including anti-inflammatory actions (88) and a strongly hypothesized positive effect on the cell-mediated immunity (89, 90)], especially on immune cells from the myeloid compartment (91). Although no statistical difference in prealbumin values was observed between the Post-V penguin groups, the significant reduction of Pre-V *vs.* Post-V prealbumin levels in the unvaccinated group may be indicative of lower immunity against *Aspergillus* sp., linked to lower cell-mediated immunity compared to vaccinated penguins. Whether the probiotic strain of *E. coli* Nissle has a positive effect on myeloid immune cells in penguins deserves further research.

## Conclusion

This study confirms that the oral use of the probiotic strain of *E. coli* Nissle is safe in the Humboldt penguin. The immune response to α-Gal in these animals is closely related to that of *E. coli*. The tested oral vaccine did not produce a significant increase in anti-α-Gal Abs as we suspect that *E. coli* Nissle expresses α-Gal at a cellular location that is not accessible to the penguin immune system. Nevertheless, a positive effect on cell-mediated immunity of vaccinated penguins can be hypothesized, as suggested by the significant decrease of the pre-albumin fraction only in the placebo group. Moreover, the excellent gut colonization property of *E. coli* Nissle and stable and long-term antigen expression (92) render this strain an ideal candidate for an oral probiotic-based vaccine. However, further studies are needed to evaluate the positive effect of *E. coli* Nissle as an oral probiotic on myeloid immune cells in the Humboldt penguin.

## Data Availability Statement

The datasets presented in this study can be found in online repositories. The names of the repository/repositories and accession number(s) can be found below: https://www.ncbi.nlm.nih.gov/, PRJNA808259.

## Ethics Statement

The animal study was reviewed and approved by an ethics committee composed of veterinarians, director and keepers from ZooParc de Beauval. The study did not involve harm or cruelty to the animals. All the procedures were performed by specialized veterinary personnel. If stress was noticed by the veterinarian during manual restraint, the animal was released for a few minutes and soft restraint by an experienced keeper was preferred afterwards. Animals were remotely closely monitored for at least 30 minutes after being released in their enclosure.

## Author Contributions

MT, AC-C, BM, AL, AH, VR-C, and LGB-H conceived the study. MT, LM-H and MNA performed the experiments and acquired the data. AW-C, and DO performed the microbiome analyses. AC-C and MT analyzed the data. MT and AL performed the CT scans and blood samplings. HG, and YR read and scored the CT scans. GD, and J-BD performed the 3-hydroxybutyrate analyses. AC-C, LM-H, AW-C, and MT prepared the figures. MT, AC-C, and DO supervised the work. AC-C, VR-C, and JF contributed with reagents and other resources. AC-C, MT, and LM-H drafted the first version of the manuscript. AW-C and LM-H edited and corrected the manuscript. All authors revised and accepted the last version of the manuscript.

## Funding

UMR BIPAR is supported by the French Government’s Investissement d’Avenir program, Laboratoire d’Excellence “Integrative Biology of Emerging Infectious Diseases” (grant no. ANR-10-LABX-62-IBEID). AW-C is supported by Programa Nacional de Becas de Postgrado en el Exterior “Don Carlos Antonio López” (grant no. 205/2018).

## Conflict of Interest

The authors declare that the research was conducted in the absence of any commercial or financial relationships that could be construed as a potential conflict of interest.

## Publisher’s Note

All claims expressed in this article are solely those of the authors and do not necessarily represent those of their affiliated organizations, or those of the publisher, the editors and the reviewers. Any product that may be evaluated in this article, or claim that may be made by its manufacturer, is not guaranteed or endorsed by the publisher.

## Supplementary Material

Supplementary Table S1 Clinical findings and suspicion of aspergillosis (from none to strong suspicion) in ten penguins from which serum samples were used to establish the competitive ELISA assay.

Supplementary Table S2 Relative abundance of bacterial taxa found in the microbiota of Humboldt penguin.

Supplementary Table S3 3-OHB plasma levels in study penguins.

Supplementary Table S4 CT scan scorings of study penguins.

Supplementary Figure S1 Contribution of bacterial taxa to α1,3GT genes in the microbiome of penguins. Sankey diagram showing the presence of α1,3GT genes (i.e., K02450, K02847, K03275, K03279, K03276, K03278) in the microbiome of various penguin species, as well as the bacterial genera harboring these genes in each host. The presence and the contribution of taxa to α1,3GT genes were predicted from 16S rRNA data using PICRUSt2 and the α1,3GT genes were annotated using the KEGG orthologs (KO) database as reference. Nodes represent penguin species (left column), functional genes (middle column) and bacterial taxa (right column). The edges represent the connection between individual host, α1,3GT genes and contributing taxon. The contribution of each taxon to different α1,3GT genes is represented proportionally by the size of the edges. K02450: gspA, general secretion pathway protein A; K02847: *waaL*, *rfaL*, O-antigen ligase [EC:2.4.1.-]; K03275: *waaO*, *rfaI*, UDP-glucose:(glucosyl)LPS alpha-1,3-glucosyltransferase [EC:2.4.1.-]; K03279: *waaJ*, *rfaJ*, UDP-glucose:(galactosyl)LPS alpha-1,2-glucosyltransferase [EC:2.4.1.58]; K03276: *waaR*, *waaT*, *rfaJ*, UDP-glucose/galactose:(glucosyl)LPS alpha-1,2-glucosyl/galactosyltransferase [EC:2.4.1.-]; K03278: waaI, rfaI, UDP-D-galactose:(glucosyl)LPS alpha-1,3-D-galactosyltransferase [EC:2.4.1.44].

Supplementary Figure S2 Recognition of chicken and penguin IgY by anti-turkey and anti-chicken IgY in a western blot assay. Total IgY was purified from chicken and penguin sera. Purified chicken and penguin IgY was run in an SDS-PAGE and transferred to membrane for the western blot reaction using anti-turkey or anti-chicken IgY. Anti-turkey IgY recognizes several bands in chicken and penguin IgY, while anti-chicken IgY recognizes only one band in chicken and penguin IgY.

Supplementary Figure S3 Levels of plasma proteins after oral administration of *E. coli* Nissle. The percentage of prealbumin **(A)**, albumin **(B)**, α1 **(C)**, α2 **(D)**, β1 **(E)**, β2 **(F)**, and γ **(G)** -globulins concentrations and the albumin/globulin (H) ratio in penguins were compared within *E. coli* Nissle (red dots) and placebo (black dots) groups before (Pre-V) and after (Post-V) treatment. Individual values are shown and were compared by paired student’s *t*-test (* *p* < 0.05; ns: not significant, 1 experiment, n = 9 in the *E. coli* Nissle group and n = 8 in the placebo group).
