# Supplementary material for: Assessment of the Safety and Efficacy of an Oral Probiotic-Based Vaccine Against Aspergillus Infection in Captive-Bred Humboldt Penguins (Spheniscus humboldti)
Source: Front Immunol. 2022 May 13;13:897223. doi: 10.3389/fimmu.2022.897223 (PMC9137413; doi:10.3389/fimmu.2022.897223)
Supplement: Supplementary Table S1 — Clinical findings and suspicion of aspergillosis (from none to strong suspicion) in ten penguins from which serum samples were used to establish the competitive ELISA assay. [file Table_1.doc]

Supplementary Table S1. Clinical findings and suspicion of aspergillosis (from none to strong) in ten penguins from which serum samples were used to establish the competitive ELISA assay.

| Group | Penguin n° | Sex | Date of sampling (frozen serum) | Clinical findings | Suspicion of aspergillosis |
| --- | --- | --- | --- | --- | --- |
| Preliminary study:  test of the competitive ELISA assay | 1 | F | 16/10/2017 | Suspicion of ocular aspergillosis on 29/05/2016 | Mild |
|  | 2 | M | 16/10/2017 | Suspicion of respiratory aspergillosis on 10/09/2016 | Mild |
|  | 3 | F | 16/10/2017 | Clinically healthy | None |
|  | 4 | F | 16/10/2017 | Suspicion of ocular aspergillosis on 18/07/2017 | Mild |
|  | 5 | F | 16/10/2017 | Clinically healthy | None |
|  | 6 | F | 16/10/2017 | Clinically healthy | None |
|  | 7 | M | 16/10/2017 | Clinically healthy | None |
|  | 8 | M | 16/10/2017 | Clinically healthy | None |
|  | 9 | M | 15/08/2020 | Mild, recurrent bilateral corneal opacification | Mild |
|  | 10 | F | 27/10/2020 | Suspicion of pulmonary aspergillosis (granuloma seen on the CT Scan) | Strong |
