# Supplementary material for: Assessment of the Safety and Efficacy of an Oral Probiotic-Based Vaccine Against Aspergillus Infection in Captive-Bred Humboldt Penguins (Spheniscus humboldti)
Source: Front Immunol. 2022 May 13;13:897223. doi: 10.3389/fimmu.2022.897223 (PMC9137413; doi:10.3389/fimmu.2022.897223)
Supplement: Supplementary Table S4 — CT scan scorings of study penguins. [file Table_4.docx]

**Supplementary Table S3. CT scan scorings of study penguins.**

|  |  |  | **Right lung nodules** | **Left lung nodules** | **Air sac nodules** | | **Air sac plaques** | | **Trachea nodules** | | | | | **Trachea plaques** |
| --- | --- | --- | --- | --- | --- | --- | --- | --- | --- | --- | --- | --- | --- | --- |
|  | **Penguin n°** | **CT exam n° (date)** | **Yes = 1 ; No = 0** | | | | | | | | | | | |
| Vaccine | 1 | 23 (17/12/21) | 0 | 0 | 0 | | | 0 | | 0 | | 0 | | |
|  |  | 24 (31/03/21° | 0 | 0 | 0 | | | 0 | | 0 | | 0 | | |
|  | 2 | 11 (17/12/20) | 0 | 0 | 0 | | | 0 | | 0 | | 0 | | |
|  |  | 12 (31/03/21) | 0 | 0 | 0 | | | 0 | | 0 | | 0 | | |
|  | 3 | 25 (17/12/20) | 0 | 0 | 0 | | | 0 | | 0 | | 0 | | |
|  |  | 26 (31/03/21) | 0 | 0 | 0 | | | 0 | | 0 | | 0 | | |
|  | 4 | 27 (16/12/20) | 0 | 0 | 0 | | | 0 | | 0 | | 0 | | |
|  |  | 28 (31/03/20) | 0 | 0 | 0 | | | 0 | | 0 | | 0 | | |
|  | 5 | 21 (16/12/20) | 0 | 0 | 0 | | | 0 | | 0 | | 0 | | |
|  |  | 22 (31/03/21) | 0 | 0 | 0 | | | 0 | | 0 | | 0 | | |
|  | 6 | 3 (16/12/20) | 0 | 0 | 0 | | | 0 | | 0 | | 0 | | |
|  |  | 4 (31/03/21) | 0 | 0 | 0 | | | 0 | | 0 | | 0 | | |
|  | 7 | 29 (17/12/20) | 0 | 0 | 0 | | | 0 | | 0 | | 0 | | |
|  |  | 30 (31/03/21) | 0 | 0 | 0 | | | 0 | | 0 | | 0 | | |
|  | 8 | 31 (17/12/20) | 0 | 0 | 0 | | | 0 | | 0 | | 0 | | |
|  |  | 32 (31/03/21) | 0 | 0 | 0 | | | 0 | | 0 | | 0 | | |
|  | 9 | 19 (1712/20) | 0 | 0 | 0 | | | 0 | | 0 | | 0 | | |
|  |  | 20 (31/03/21) | 0 | 0 | 0 | | | 0 | | 0 | | 0 | | |
|  | 10 | 13 (16/12/20) | 0 | 0 | 0 | | | 0 | | 0 | | 0 | | |
|  |  | 14 (31/03/21) | 0 | 0 | 0 | | | 0 | | 0 | | 0 | | |
| Placebo | 11 | 17 (16/12/20) | 0 | 0 | 0 | | | 0 | | 0 | | 0 | | |
|  |  | 18 (30/03/21) | 0 | 0 | 0 | | | 0 | | 0 | | 0 | | |
|  | 12 | 33 (16/12/20) | 0 | 0 | 0 | | | 0 | | 0 | | 0 | | |
|  |  | 34 (31/03/21) | 0 | 0 | 0 | | | 0 | | 0 | | 0 | | |
|  | 13 | 1 (16/12/20) | 0 | 0 | 0 | | | 0 | | 0 | | 0 | | |
|  |  | 2 (31/03/21) | 0 | 0 | 0 | | | 0 | | 0 | | 0 | | |
|  | 14 | 9 (16/12/20) | 0 | 0 | 0 | | | 0 | | 0 | | 0 | | |
|  |  | 10 (31/03/21) | 0 | 0 | 0 | | | 0 | | 0 | | 0 | | |
|  | 15 | 5 (17/12/20) | 0 | 0 | 0 | | | 0 | | 0 | | 0 | | |
|  |  | 6 (31/03/21)* | - | - | - | | | - | | - | | - | | |
|  | 16 | 35 (16/12/20) | 0 | 0 | 0 | | | 0 | | 0 | | 0 | | |
|  |  | 36 (31/03/21) | 0 | 0 | 0 | | | 0 | | 0 | | 0 | | |
|  | 17 | 7 (17/12/20) | 0 | 0 | 0 | | | 0 | | 0 | | 0 | | |
|  |  | 8 (31/03/21)* | - | - | - | | | - | | - | | - | | |
|  | 18 | 15 (20/12/20) | 0 | 0 | 0 | | 0 | | 0 | | | | | 0 |
|  |  | 16 (31/03/21) | 0 | 0 | 0 | | 0 | | 0 | | | | | 0 |
|  | 19 | 37 (16/12/20) | 0 | 0 | 0 | 0 | | | | | 0 | | 0 | |
|  |  | 38 (31/03/21) | 0 | 0 | 0 | 0 | | | | | 0 | | 0 | |
|  | 20 | 39 (17/12/20) | 0 | 0 | 0 | 0 | | | | | 0 | | 0 | |
|  |  | 40 (31/03/20) | 0 | 0 | 0 | 0 | | | | | 0 | | 0 | |

Interpretation of CT scans n° 6 (penguins n°15) and 8 (penguins n°17) were not possible due to excessive motion artifact.
