# Supplementary figures and images for: Assessment of the Safety and Efficacy of an Oral Probiotic-Based Vaccine Against Aspergillus Infection in Captive-Bred Humboldt Penguins (Spheniscus humboldti)
Source: Front Immunol. 2022 May 13;13:897223. doi: 10.3389/fimmu.2022.897223 (PMC9137413; doi:10.3389/fimmu.2022.897223)

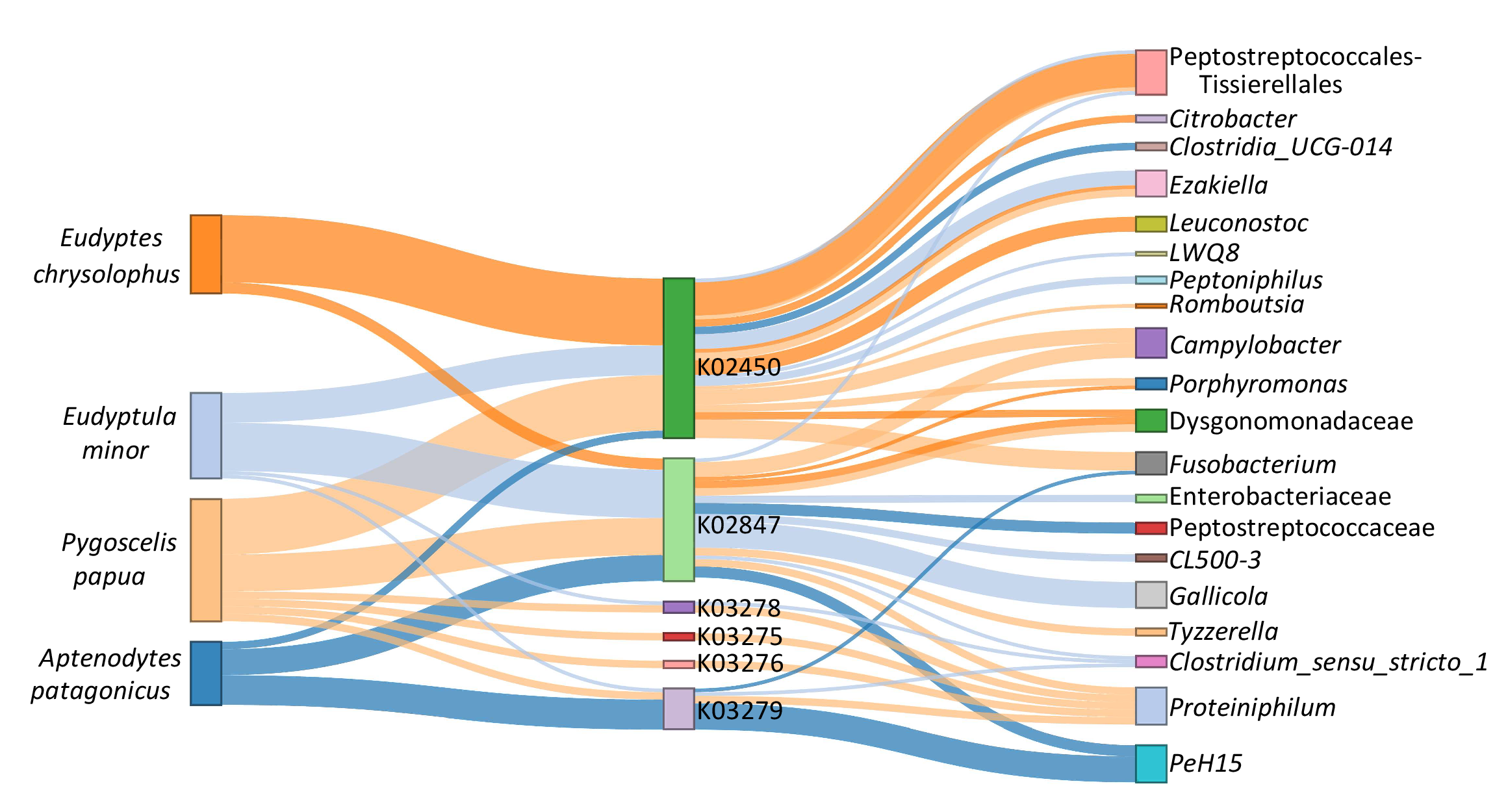

Supplement: Supplementary Figure S1 — Contribution of bacterial taxa to α1,3GT genes in the microbiome of penguins. Sankey diagram showing the presence of α1,3GT genes (i.e., K02450, K02847, K03275, K03279, K03276, K03278) in the microbiome of various penguin species, as well as the bacterial genera harboring these genes in each host. The presence and the contribution of taxa to α1,3GT genes were predicted from 16S rRNA data using PICRUSt2 and the α1,3GT genes were annotated using the KEGG orthologs (KO) database as reference. Nodes represent penguin species (left column), functional genes (middle column) and bacterial taxa (right column). The edges represent the connection between individual host, α1,3GT genes and contributing taxon. The contribution of each taxon to different α1,3GT genes is represented proportionally by the size of the edges. K02450: gspA, general secretion pathway protein A; K02847: waaL, rfaL, O-antigen ligase [EC:2.4.1.-]; K03275: waaO, rfaI, UDP-glucose:(glucosyl)LPS alpha-1,3-glucosyltransferase [EC:2.4.1.-]; K03279: waaJ, rfaJ, UDP-glucose:(galactosyl)LPS alpha-1,2-glucosyltransferase [EC:2.4.1.58]; K03276: waaR, waaT, rfaJ, UDP-glucose/galactose:(glucosyl)LPS alpha-1,2-glucosyl/galactosyltransferase [EC:2.4.1.-]; K03278: waaI, rfaI, UDP-D-galactose:(glucosyl)LPS alpha-1,3-D-galactosyltransferase [EC:2.4.1.44]. [file Image_1.tiff]

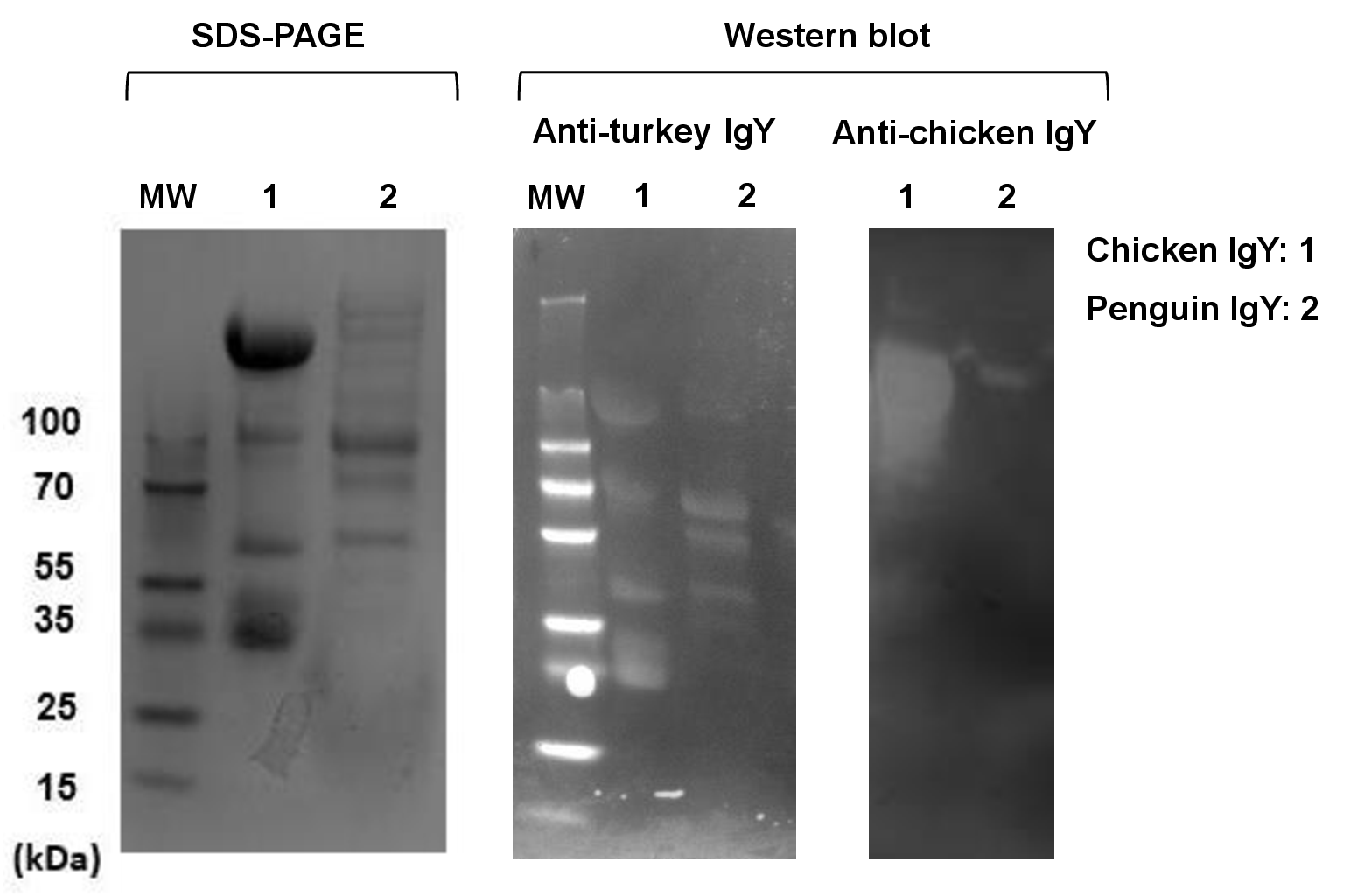

Supplement: Supplementary Figure S2 — Recognition of chicken and penguin IgY by anti-turkey and anti-chicken IgY in a western blot assay. Total IgY was purified from chicken and penguin sera. Purified chicken and penguin IgY was run in an SDS-PAGE and transferred to membrane for the western blot reaction using anti-turkey or anti-chicken IgY. Anti-turkey IgY recognizes several bands in chicken and penguin IgY, while anti-chicken IgY recognizes only one band in chicken and penguin IgY. [file Image_2.tiff]

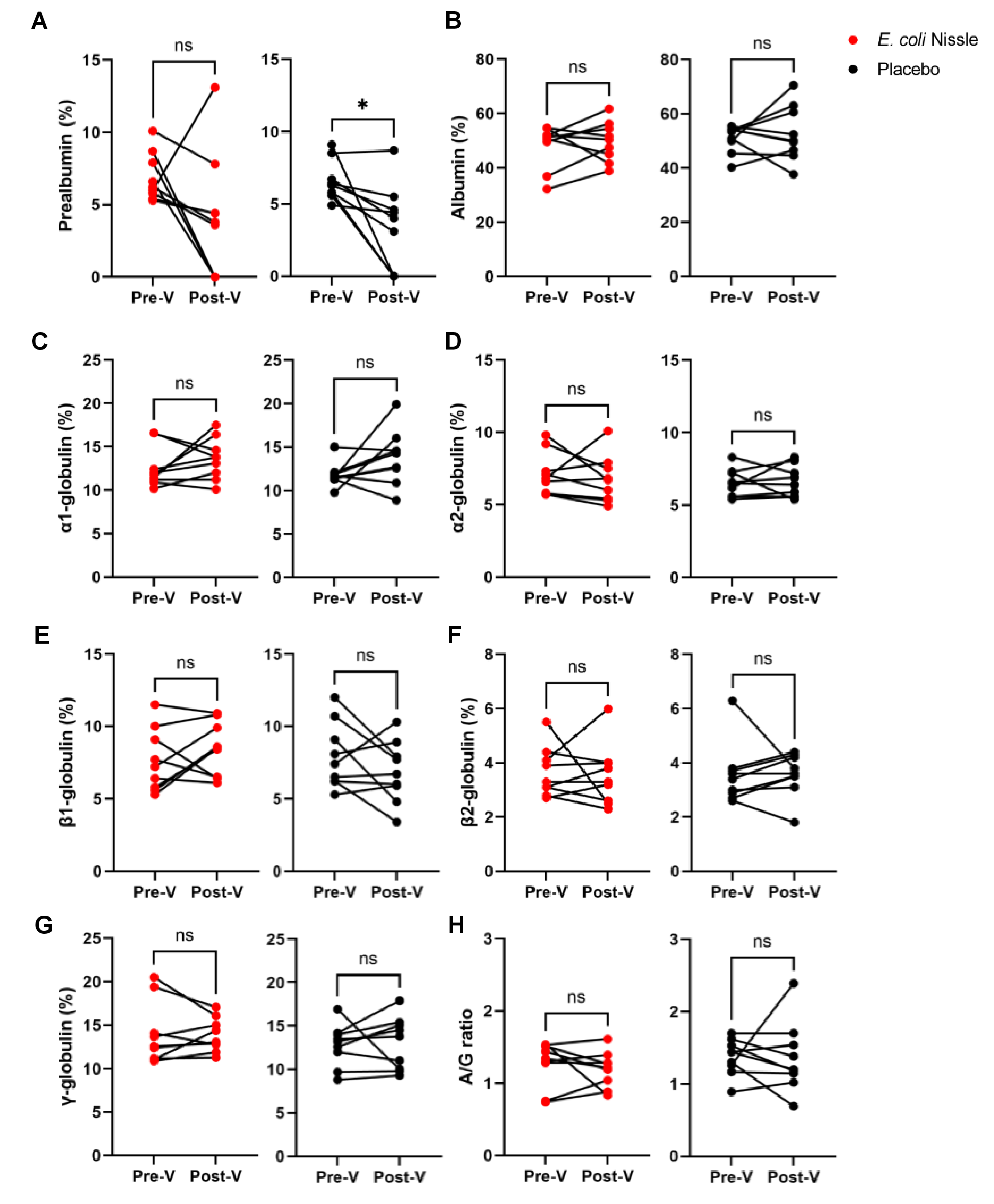

Supplement: Supplementary Figure S3 — Levels of plasma proteins after oral administration of E. coli Nissle. The percentage of prealbumin (A), albumin (B), α1 (C), α2 (D), β1 (E), β2 (F), and γ (G) -globulins concentrations and the albumin/globulin (H) ratio in penguins were compared within E. coli Nissle (red dots) and placebo (black dots) groups before (Pre-V) and after (Post-V) treatment. Individual values are shown and were compared by paired student’s t-test (* p < 0.05; ns: not significant, 1 experiment, n = 9 in the E. coli Nissle group and n = 8 in the placebo group). [file Image_3.tiff]
